# Quality Properties of Dry-Aged Beef (Hanwoo Cattle) Crust on Pork Patties

**DOI:** 10.3390/foods11152191

**Published:** 2022-07-23

**Authors:** Jeong-Ah Lee, Hack-Youn Kim, Kuk-Hwan Seol

**Affiliations:** 1 National Institute of Animal Science, Rural Development Administration, Wanju 55365, North Jeolla, Korea; 2970703@naver.com; 2 Department of Animal Resources Science, Kongju National University, Yesan 32439, Chungnam, Korea

**Keywords:** crust, dry-aged, natural flavor enhancer, patties, Hanwoo cattle

## Abstract

This study evaluated the effects of crust derived from dry-aged beef (Hanwoo cattle) on the quality of pork patties. Pork patty samples were prepared with different amounts of crust (0—control, 1, 2, and 3%). The protein, fat, and ash contents in the crust samples were significantly higher than those in the control sample (*p* < 0.05). The CIE b^*^ value of uncooked pork patties with crust added was significantly lower than that of the control patties (*p* < 0.05). The pH and CIE L* values of uncooked patty batter samples decreased with increasing concentrations of crust (*p* < 0.05). However, the viscosity increased proportionally with an increase in crust (*p* < 0.05). Samples containing 3% crust showed significantly higher uncooked and cooked CIE a*, water-holding capacity, cooking yield, and shear force than the control sample (*p* < 0.05). Moreover, samples containing 2% and 3% crust showed significantly lower diameter and thickness reductions than those of the control sample (*p* < 0.05). The sensory evaluation conferred by the crust was significantly higher than that of the control sample (*p* < 0.05). Overall, our results suggest that pork patties supplemented with 3% crust have improved properties.

## 1. Introduction

In Korea, there has been an increase in consumer preference for Hanwoo dry-aged beef, a high-quality meat product with unique flavor and tenderness [1]. Dry aging meat occurs in an aerobic environment under specific humidity and temperature conditions for several weeks. This dry-aged meat develops characteristics that are different from those of unaged meat. Continuous proteolysis tenderizes the meat and produces a softer texture [2], while the byproducts of proteolysis and lipolysis, free amino acids and free fatty acids, respectively, add a distinctive flavor [3,4]. However, because the surface of dry-aged meat is exposed to air (under aerobic conditions), moisture is continuously evaporated and results in a hardened surface known as the crust [5,6]. The crust formed accounts for approximately 35% of dry-aged meat and is trimmed after processing as it cannot be used for roasting. As such, discarded crust is the primary reason for the low yield and high price of dry-aged meat [6].

However, non-edible crust can provide economic value. Since it has a concentrated “dry-aging flavor” owing to moisture evaporation, it can be used as a flavor enhancer [7]. Crust can also be used as a natural and bioactive food additive because it exhibits higher antioxidant and antihypertensive properties than raw, wet-aged, and edible dry-aged meat [8]. Interestingly, Park et al. [9] reported that the addition of crust to beef patties enhanced their taste, flavor, and tenderness, suggesting the potential use of crust as an additive for meat products.

Ground meat products, such as patties and sausages, are the most common form of processed meat products consumed worldwide [10,11]. Since the introduction of hamburgers in Korea in 1973, the market has expanded to include various types of ground meat products [12]. Patties are manufactured by shaping ground beef and pork combined with various ingredients that enhance sensory properties, such as flavor, juiciness, and texture [13]. Some meat products, such as patties and sausages, are popular, but consumer preferences are shifting toward health and wellness concerns. In response, researchers have generated ground meat products with improved quality via the addition of high-quality functional ingredients tailored to consumer needs [14,15,16,17,18]. Most of these functional ingredients are derived from natural sources, of which plant-based materials form the majority, and commonly unconsumed meat byproducts. Since the crust obtained from dry-aged meat is extracted from edible meat, research on its application in various products, such as products derived from cattle, must be performed to determine its suitability as an additive.

Therefore, in this study, we analyzed the physicochemical properties of the addition of crust derived from dry-aged beef (Hanwoo cattle) to pork patties.

## 2. Materials and Methods

### 2.1. Hanwoo Crust and Pork Pattie Preparation

The dry-aging and crust separation process of beef loin (Hanwoo) was as follows: six pieces of beef loin (M. longissimus dorsi) were obtained from six carcasses two days after being slaughtered (Hanwoo cattle, Korean quality grade 2, Tobawoo, Sejong, Korea). Samples were dry aged in a refrigerator (aging temperature, 4 ± 1 °C; humidity, 65 ± 5%; air velocity, 5 ± 3 m/s) for four weeks. After dry aging, the crust was collected from the outermost edge (0.5 ± 0.2 cm) of the dry-aged beef loin, then lyophilized in a freeze dryer (FDU-1110, Eyela, Tokyo, Japan) at −70 °C for 48 h. Lastly, the lyophilized crust derived from Hanwoo beef loin was pulverized using a mixer (MQ5135, Braun, Kronberg im Taunus, Germany) and used in this study.

Pork patties were formulated with various amounts of crust (Table 1). To prepare the meat batter samples, pork hind leg meat and back fat were purchased from a local market (I home meat, Seoul, Korea). Pork hind leg meat and back fat were cut and ground using a grinder (PA-82, Mainca, Barcelona, Spain) attached to a 3 mm plate. Next, the ground meat (70%) and back fat (15%) were mixed with iced water (15%), garlic powder (0.5%), sugar (1%), nitrite pickling salt (1.2%; nitrite content: 6000 ppm), onion powder (0.5%), and crust at different concentrations (0, 1, 2, and 3%). Each mixture was used to prepare pork patties (100 g each) using a pressure device. Pork patties were thermally processed in a chamber (10.10ESI/SK, Alto Shaam, Menomonee Falls, WI, USA) at 80 °C for 30 min.

### 2.2. Determination of the Proximate Composition

The proximate composition of the cooked patties was determined according to the AOAC method [19]. The moisture, protein, fat, and ash contents were measured using 105 °C oven drying, Kjeldahl, Soxhlet, and 550 °C dry ashing methods, respectively.

### 2.3. Determination of the pH Values

To measure the pH of the cooked pork patties, 5 g of each sample was placed in a conical tube with 20 mL of distilled water. The samples were homogenized using an Ultra-Turrax homogenizer (HMZ-20DN, Poolim Tech, Seongnam, Korea) at 8000 rpm for 1 min. The pH values of the mixtures of samples were determined using a glass electrode pH meter (Model 340; Mettler-Toledo, Schwerzenbach, Switzerland).

### 2.4. Determination of the Color of Samples

The surface area color of the cooked patties was measured using a colorimeter (CR-10. Minolta, Tokyo, Japan) for CIE L* (lightness), CIE a* (redness), and CIE b* (yellowness) values. A white standard plate was used as a reference (CIE L*: +97.83; CIE a*: −0.43; CIE b*: +1.98).

### 2.5. Determination of the Water-Holding Capacity (WHC)

The WHC was determined according to a method adapted from [20]. Briefly, 5 g of each sample was placed inside a conical tube containing cotton and a filter paper. Samples were centrifuged (Supra R22, Hanil, Gimpo, Korea) at 1092× *g*, 4 °C for 10 min. After centrifugation, the WHC (as per the weight of the water-drained sample) was calculated using the following formula

(1) $$\mathrm{WHC}\;\left(\%\right)=\frac{A-B}{B}\times 100\;$$

A: Sample mass before centrifugation (*g*) × water content of sample (%)) ÷ 100.

B: Sample mass before centrifugation (*g*) – sample mass after centrifugation (*g*).

### 2.6. Determination of the Cooking Yield

The cooking yield of patty samples was determined by calculating the mass before and after cooking, as follows

(2) $$\mathrm{Cooking}\;\mathrm{yield}\;\left(\%\right)=\frac{\mathrm{Sample}\;\mathrm{mass}\;\mathrm{after}\;\mathrm{cooking}\;\left(g\right)}{\mathrm{Sample}\;\mathrm{mass}\;\mathrm{before}\;\mathrm{cooking}\;\left(g\right)}\times 100$$

### 2.7. Determination of Viscosity

The apparent viscosity of the patty batter samples was measured using a viscometer (Merlin VR, Rheosys, Hamilton Township, NJ, USA) equipped with a 30-mm cone and a 25-mm co-axial cylinder. Measurements were conducted for 60 s at 20 °C at a head speed of 20 rpm. The measured apparent viscosity values were expressed in Pa·s.

### 2.8. Determination of the Diameter and Thickness Reduction Ratio

The diameter and thickness reduction ratios were determined by measuring the diameter and thickness of the patties before and after cooking using Vernier calipers (CD-15APX; Mitutoyo Co., Kawasaki-shi, Japan). Values are expressed as percentages, according to the following formulas

(3) $$\mathrm{Diameter}\;\mathrm{reduction}\;\mathrm{ratio}\;\left(\%\right)=\frac{\mathrm{Diameter}\;\mathrm{before}\;\mathrm{cooking}\;\left(\mathrm{mm}\right)-\mathrm{Diameter}\;\mathrm{after}\;\mathrm{cooking}\;\left(\mathrm{mm}\right)}{\mathrm{Diameter}\;\mathrm{before}\;\mathrm{cooking}\;\left(\mathrm{mm}\right)}\times 100$$

(4) $$\mathrm{Thickness}\;\mathrm{reduction}\;\mathrm{ratio}\;\left(\%\right)=\frac{\mathrm{Diameter}\;\mathrm{before}\;\mathrm{cooking}\;\left(\mathrm{mm}\right)-\mathrm{Diameter}\;\mathrm{after}\;\mathrm{cooking}\;\left(\mathrm{mm}\right)}{\mathrm{Diameter}\;\mathrm{before}\;\mathrm{cooking}\;\left(\mathrm{mm}\right)}\times 100$$

### 2.9. Determination of the Shear Force

The shear force was determined for each sample by attaching them to 2.0 cm^3^ sample blocks and evaluated using a Texture Analyzer with an attached v-blade (head speed, 2.0 mm/s; distance, 22.0 mm; downforce, 5 g; TA 1, Ametek, Largo, FL, USA), and the measurements were expressed in Newtons (N).

### 2.10. Sensory Evaluation

The sensory evaluation of this study was approved by the Ethics Committee of Kongju National University (Authority IRB No: KNU 2020-15), and sensory evaluation was performed by ten sensory panelists. The panelists were trained with commercially available pork patties for 7 d (1 h session per day) to ensure familiarization with the sensory properties of pork patties. The sensory evaluations were color, flavor, tenderness, juiciness, and overall acceptability, and the samples were scored using a 10-point descriptive scale.

### 2.11. Statistical Analysis

All the experimental data were collected from at least three independent trials. The data were statistically analyzed using analysis of variance for all variables, followed by Duncan’s multiple range test (*p* < 0.05), and the general linear model in SAS software (version 9.3, SAS Institute, Cary, NC, USA). Values are expressed as the mean ± SD.

## 3. Results and Discussion

### 3.1. Effect of Lyophilized Crust Derived from Dry-Aged Beef (Hanwoo) Supplementation on Proximate Composition

The proximate composition of cooked pork patties, according to the amount of crust derived from dry-aged beef (Hanwoo), is shown in Table 2. Crust addition significantly lowered the moisture content compared with the control samples (*p* < 0.05).

In contrast, protein and fat contents significantly increased with increasing crust content compared with the control samples (*p* < 0.05). Campbell et al. [21] and Lee et al. [22] reported that dry-aged meat has higher protein and ash contents, which are major components of muscle tissue, because a large amount of moisture evaporates during the aging process. As such, our results are consistent with existing research demonstrating that increasing the amount of crust increases the protein and ash contents [23]. Crust has high protein and fat content as it originates from the surface of Hanwoo beef, and it contains almost no moisture owing to dry aging. Similarly, when lyophilized protein additives are added to meat products, protein, fat, and ash contents increase as the moisture content decreases [24]. Therefore, it is believed that meat products with high protein, fat, and ash content can be obtained via the addition of crust.

### 3.2. Effect of Lyophilized Crust Derived from Dry-Aged Beef (Hanwoo) Supplementation on pH and Color

Table 3 shows the pH and CIE color of uncooked and cooked pork patties with crust derived from dry-aged beef (Hanwoo). The pH of the uncooked pork patties significantly decreased as the amount of crust increased (*p* < 0.05). The pH of the cooked patties also tended to decrease with increasing crust content. In addition, pork patties containing 3% crust showed a significantly lower pH than the control sample (*p* < 0.05). Therefore, we assumed that the pH of pork patties is affected by this crust addition because the pH of meat products can vary with the pH range of additive(s) [25]. Because the pH of the crust was 5.67, it is not surprising that higher amounts of crust led to greater decreases in the pH. Interestingly, the pH values were higher in cooked versus uncooked samples, which was consistent with studies reporting that the pH increases as basic active species (e.g., imidazolium present in the amino acid histidine) are exposed to the external environment in the context of heat-induced protein denaturation [26].

With respect to color, the lightness of the uncooked and cooked samples tended to decrease as the crust content increased. In addition, lightness before cooking significantly decreased as the amount of crust increased (*p* < 0.05). The redness of uncooked and cooked patties tended to increase with increasing crust content, and the highest value was observed in patties containing 3% crust (*p* < 0.05). The yellowness of the uncooked samples significantly decreased with increasing crust added compared with the control sample (*p* < 0.05). The results in this work are similar to those reported in a study by Kim et al. [27]: lightness and yellowness decreased as redness increased due to the increase in the myoglobin content (red-colored component) present in the meat additive. As aging time increases, myoglobin in meat is oxidized to metmyoglobin, which is a discolored brown. This is a result of the relative increase in myoglobin content as moisture evaporates [28]. Nevertheless, the crust color is similar to that of typical meat products. Overall, we showed that the change in color was affected by the lightness (37.82), redness (12.87), and yellowness (2.08) of the Hanwoo crust. Finally, the yellowness of cooked samples is likely to be less affected in pork patties because the proteins contained in the crust are heat-denatured by the Maillard reaction [29].

### 3.3. Effect of Lyophilized Crust Derived from Dry-Aged Beef (Hanwoo) Supplementation on Water-Holding Capacity (WHC) and Cooking Yield

**Figure 1:**
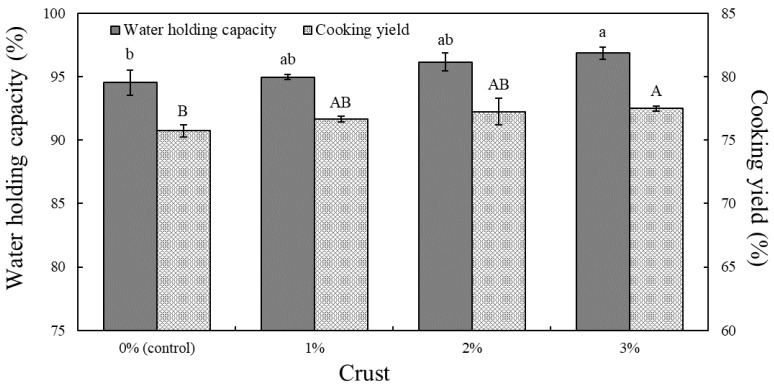
Water-holding capacity (WHC) and cooking yield of pork patties formulated with various levels of crust derived from dry-aged beef (Hanwoo). ^a,b,A,B^ represent statistically significant differences comparing the same color bar graphs (*p* < 0.05).

The WHC and cooking yield of meat products depend on the cross-linked state of salt-soluble proteins, water molecules, and fat globules [30], which are affected by the structure, amount, protein content, and physical properties [31]. Figure 1 shows the WHC of uncooked pork patty batter and cooking yield of pork patties with different crust levels derived from dry-aged beef (Hanwoo).

The WHC and cooking yield tended to increase with an increasing amount of crust; in particular, significantly higher values were obtained in pork patties containing 3% crust than in the control samples (*p* < 0.05). The WHC change is probably due to the rehydration of the lyophilized crust, which increased with the amount of crust added [32]. In a similar study, Choi et al. [33] added lyophilized mealworms to pork patties and noted that the WHC increased because of reduced cooking loss. The high cooking yield of samples containing crust is attributed to stronger bonds between protein molecules and water molecules [34], resulting in a lower cooking loss. The lyophilized crust with a high rehydration potential strengthened the bond with water molecules in the patties and improved the cooking yield and WHC, which is characteristic of highly rehydratable additives [24].

### 3.4. Effect of Lyophilized Crust Derived from Dry-Aged Beef (Hanwoo) Supplementation on Viscosity

**Figure 2:**
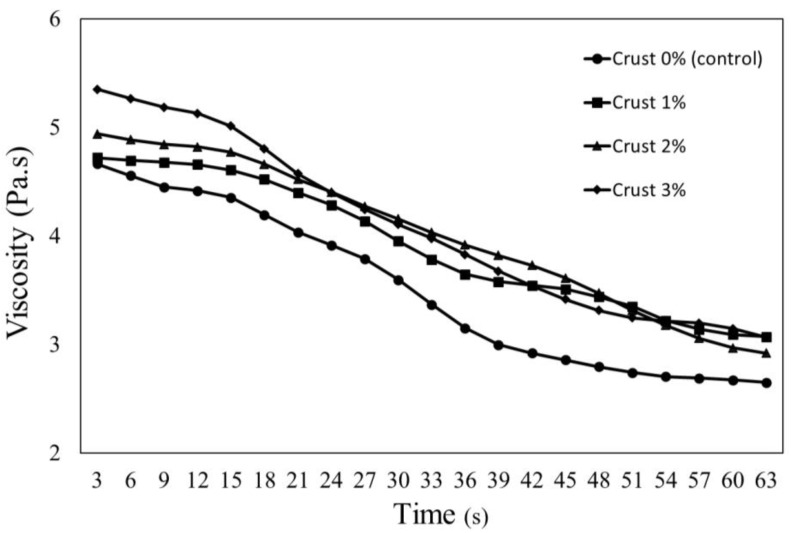
Apparent viscosity of pork patties formulated with various levels of crust derived from dry-aged beef (Hanwoo).

The viscosity of uncooked pork patty batter containing crust derived from dry-aged beef (Hanwoo) is shown in Figure 2. The apparent viscosity increased with an increasing amount of crust, and all samples containing crust showed higher apparent viscosities compared with the control samples. This viscosity increase was attributed to the enhanced WHC of the added crust. Similar to these results, June et al. [35] demonstrated that the addition of lyophilized protein-type walnuts to a non-Newtonian fluid led to an increase in viscosity.

The viscosity of uncooked ground meat is affected by physical properties, such as WHC, protein shrinkage, and solubility, and it exhibits a flow curve characteristic of a non-Newtonian fluid [36]. The apparent viscosity of emulsified patties also decreased with increasing rotation time, and this behavior is characteristic of thixotropic fluids; protein molecules (e.g., in ground meat) form a more ordered arrangement with increasing rotation time, leading to a gradual decrease in viscosity [37].

### 3.5. Effect of Lyophilized Crust Derived from Dry-Aged Beef (Hanwoo) Supplementation on Diameter and Thickness Reduction Ratios

**Figure 3:**
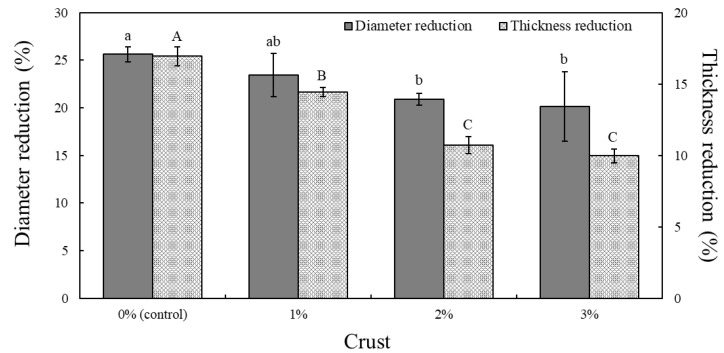
Diameter and thickness reduction ratios of pork patties formulated with various levels of crust derived from dry-aged beef (Hanwoo). ^a,b,A–C^ Represent statistically significant differences comparing the same color bar graphs (*p* < 0.05).

The diameter and thickness reduction ratios of the cooked pork patties with various crust levels are shown in Figure 3. The diameter and thickness reduction ratios were significantly lower in pork patties containing 2% and 3% crust than in the control samples (*p* < 0.05).

These results were consistent with previous findings that showed that non-meat proteins, such as wheat germ and soy proteins, added to patties resulted in lower cooking loss and shrinkage [38]. Our results were also similar with those of a study reporting a smaller reduction in the diameter and thickness of pork patties prepared with isolated soy protein that increased the bond strength between water and fat, thus minimizing size loss during cooking [12]. The diameter and thickness reduction ratios in pork patties are also related to organoleptic properties. Berry and Leddy [39] reported that patties with a higher cooking loss also showed poorer sensory properties. Therefore, the addition of crust enhanced the WHC and cooking yield of pork patties, thereby suppressing their size reduction during cooking. Notably, this is a positive factor concerning the hydration properties and palatability of meat products.

### 3.6. Effect of Lyophilized Crust Derived from Dry-Aged Beef (Hanwoo) Supplementation on Shear Force

**Figure 4:**
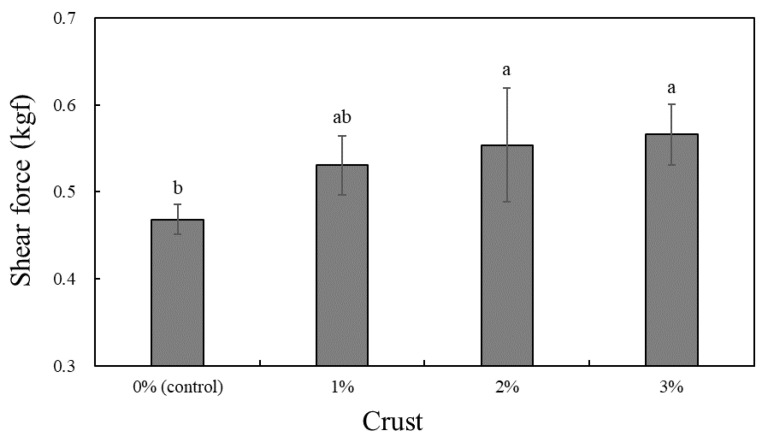
Shear force of pork patties formulated with various levels of crust derived from dry-aged beef (Hanwoo). ^a,b^ Represent statistically significant differences comparing the same color bar graphs (*p* < 0.05).

Figure 4 shows the shear force measurements of pork patties containing crusts derived from dry-aged beef (Hanwoo). The shear force of pork patties with crust tended to increase, and samples that contained 2% or 3% crust showed significantly higher values (*p* < 0.05).

Lu and Chen [40] noted that improved binding ability in meat is enhanced both by protein–protein and protein–fat binding strength, which thereby increases the shear force. Similarly, we believe that the shear force of patties increased owing to the enhanced binding strength between proteins and fat in the pork patty batter mixture with added crust. These results are also in agreement with a study on hamburger patties supplemented with lyophilized tofu powder (mainly globulin protein) that increased patty hardness with increasing levels of powder [41]. Sample hardness containing isolated soy protein and dietary fiber was also shown to be higher in a study by Choi et al. [42]. In addition, this study also reported that isolated soy and wheat fiber proteins affect the water-holding capacity, emulsifying capacity, gel-forming capacity, and adhesion between particles. Furthermore, the textural properties of meat products are affected by the condition of the raw meat and the type of additive [43,44]. Likewise, we believe that the shear force of patties increased with increasing crust content, owing to both the increase in protein content and the decrease in moisture content in patty composition.

### 3.7. Sensory Evaluation

Sensory evaluation of pork patties containing crust derived from dry-aged beef (Hanwoo) is presented in Table 4.

The sensory evaluation of color of 2% and 3% crust samples was significantly higher than that of the control sample (*p* < 0.05). The flavor tended to be higher as the added amount of crust increased, whereas tenderness showed no significant difference between all samples. The 2% crust sample received a significantly higher juiciness evaluation score than the control sample (*p* < 0.05). The 2% and 3% crust samples received significantly higher evaluations of overall acceptability than the control sample (*p* < 0.05). This evaluation was similar to a study by Park et al. [9], which reported that beef patties supplemented with crust obtained from dry-aged beef sirloin received higher scores for taste, flavor, juiciness, and overall acceptability compared to the control samples. As the meat protein and fat decompose during the dry-aging process, the content of inosine 5′-monophosphate disodium (IMP) increases, which is a flavor enhancer that elicits umami. Consequently, the enhanced flavor and umami were noted, and the products were considered more acceptable overall. Such differences in sensory properties depended on crust addition. Therefore, the added crust enhanced the flavor and played the role of an umami enhancer, and this added crust imparted a dry-aging flavor and enhanced organoleptic properties in these pork patties.

## 4. Conclusions

In this study, pork patties were prepared using added crust from dry-aged beef loin (Hanwoo cattle) as a flavor enhancer, and their qualities were analyzed. The experimental results demonstrated that increasing the amount of crust added to pork patties improved their physical properties, such as WHC, cooking yield, viscosity, and diameter and thickness reduction ratios. Moreover, the addition of crust to pork patties resulted in a better sensory evaluation. Therefore, the addition of 3% crust to pork patties should lead to the production of patties with better quality and enhanced flavor.

## Figures and Tables

**Table 1:** Formulation of the pork patties used in this study.

Ingredients (%)		Crust (%)			
		0 (Control)	1	2	3
Main	Pork lean meat	70	70	70	70
	Pork back fat	15	15	15	15
	Ice	15	15	15	15
Additives	NPS ^(1)^	1.2	1.2	1.2	1.2
	Sugar	1	1	1	1
	Garlic powder	0.5	0.5	0.5	0.5
	Onion powder	0.5	0.5	0.5	0.5
	Crust	0	1	2	3

^(1)^ NPS: nitrite pickling salt.

**Table 2:** Proximate composition of the pork patties formulated with various levels of crust derived from dry-aged beef (Hanwoo).

Traits	Crust (%)			
	0 (Control)	1	2	3
Moisture (%)	62.38 ± 0.67 ^a^	59.09 ± 1.66 ^b^	58.17 ± 0.11 ^b^	57.67 ± 0.23 ^b^
Protein (%)	21.20 ± 0.25 ^c^	23.02 ± 0.15 ^b^	23.33 ± 0.51 ^ab^	23.80 ± 0.12 ^a^
Fat (%)	14.67 ± 0.58 ^c^	16.50 ± 0.71 ^b^	17.67 ± 0.58 ^b^	19.33 ± 0.58 ^a^
Ash (%)	1.46 ± 0.25 ^b^	1.79 ± 0.10 ^a^	1.74 ± 0.09 ^a^	1.78 ± 0.04 ^a^

All values are expressed as mean ± SD. ^a–c^ Means on the same row with different letters are significantly different (*p* < 0.05).

**Table 3:** pH and color of pork patties formulated with various levels of crust derived from dry-aged beef (Hanwoo).

Traits			Crust (%)			
			0 (Control)	1	2	3
pH		Uncooked	6.14 ± 0.01 ^a^	6.13 ± 0.01 ^b^	6.09 ± 0.01 ^c^	6.08 ± 0.01 ^d^
		Cooked	6.31 ± 0.01 ^a^	6.31 ± 0.01 ^a^	6.27 ± 0.01 ^b^	6.25 ± 0.01 ^c^
Color	Uncooked	CIE L*	62.70 ± 1.27 ^a^	60.65 ± 1.63 ^ab^	59.50 ± 0.28 ^b^	57.65 ± 0.49 ^b^
		CIE a*	3.80 ± 0.01 ^b^	4.0 ± 0.14 ^b^	4.35 ± 0.07 ^b^	5.40 ± 0.71 ^a^
		CIE b*	14.20 ± 0.01 ^a^	13.60 ± 0.14 ^b^	11.60 ± 0.14 ^c^	11.25 ± 0.21 ^c^
	Cooked	CIE L*	67.53 ± 0.51 ^a^	63.43 ± 0.42 ^b^	62.75 ± 0.07 ^bc^	62.50 ± 0.00 ^c^
		CIE a*	4.00 ± 0.28 ^b^	4.15 ± 0.21 ^b^	4.75 ± 0.21 ^b^	5.85 ± 0.49 ^a^
		CIE b*	13.45 ± 0.35	12.40 ± 0.14	12.20 ± 0.42	11.50 ± 1.70

All values are expressed as mean ± SD. ^a–d^ Means on the same row with different letters are significantly different (*p* < 0.05).

**Table 4:** Sensory evaluation of pork patties formulated with various levels of crust derived from dry-aged beef (Hanwoo).

Traits	Crust (%)			
	0 (Control)	1	2	3
Color	8.00 ± 0.93 ^b^	8.75 ± 0.93 ^ab^	9.38 ± 0.74 ^a^	9.38 ± 0.74 ^a^
Flavor	8.13 ± 0.83 ^b^	8.63 ± 0.52 ^bc^	9.25 ± 0.46 ^ab^	9.50 ± 1.07 ^a^
Tenderness	9.00 ± 0.76	9.25 ± 0.46	9.13 ± 0.83	8.88 ± 0.64
Juiciness	8.38 ± 0.52 ^b^	9.00 ± 0.53 ^ab^	9.13 ± 0.64 ^a^	9.00 ± 0.76 ^ab^
Overall acceptability	8.00 ± 0.93 ^b^	8.81 ± 0.53 ^a^	9.44 ± 0.50 ^a^	9.38 ± 0.92 ^a^

All values are expressed as mean ± SD. ^a–c^ Means on the same row with different letters are significantly different (*p* < 0.05).

## Data Availability

All related data and methods are presented in this paper. Additional inquiries should be addressed to the corresponding author.
